# Seroprevalence and Potential Risk Factors for *Brucella* Spp. Infection in Traditional Cattle, Sheep and Goats Reared in Urban, Periurban and Rural Areas of Niger

**DOI:** 10.1371/journal.pone.0083175

**Published:** 2013-12-16

**Authors:** Abdou Razac Boukary, Claude Saegerman, Emmanuel Abatih, David Fretin, Rianatou Alambédji Bada, Reginald De Deken, Halimatou Adamou Harouna, Alhassane Yenikoye, Eric Thys

**Affiliations:** 1 Department of Livestock promotion and Management of Natural Resources, ONG Karkara, Niamey, Niger; 2 Department of Infectious and Parasitic Diseases, Research Unit in Epidemiology and Risk Analysis applied to the Veterinary Sciences (UREAR), University of Liege, Liege, Belgium; 3 Department of Biomedical Sciences, Unit of Biostatistics and Epidemiology, Institute of Tropical Medicine, Antwerp, Belgium; 4 Department of Bacteriology and Immunology, Veterinary and Agro-chemical Research Centre, Uccle, Belgium; 5 Service of Microbiology-Immunology-Infectious Pathology, Interstate School of Veterinary Sciences and Medicine, Dakar, Senegal; 6 Ministry of Livestock, Direction of Animal Health, Maradi, Niger; 7 Faculty of Agronomy, University of Niamey, Niamey, Niger; Iowa State University, United States of America

## Abstract

**Introduction:**

In Niamey, Niger, interactions within the interface between animals, humans and the environment induce a potential risk of brucellosis transmission between animals and from animals to humans. Currently, little is known about the transmission of *Brucella* in this context.

**Results:**

5,192 animals from 681 herds were included in the study. Serum samples and hygroma fluids were collected. A household survey enabled to identify the risk factors for transmission of brucellosis. The true adjusted herd-level prevalence of brucellosis ranged between 11.2% and 17.2% and the true adjusted animal-population level prevalence was 1.3% (95% CI: 0.9–1.8%) based on indirect ELISA test for *Brucella* antibodies. Animals aged of 1–4 years were found to be more susceptible than animals less than 1 year old (Odds ratio [OR] of 2.7; 95% CI: 1.43–5.28). For cattle, the odds of brucellosis seropositivity were higher in rural compared to the periurban areas (OR of 2.8; 95% CI: 1.48–5.17) whereas for small ruminants the risk of seropositivity appeared to be higher in urban compared to periurban areas (OR of 5.5; 95% CI: 1.48–20.38). At herd level, the risk of transmission was increased by transhumance (OR of 5.4; 95% CI: 2.84–10.41), the occurrence of abortions (OR of 3.0; 95% CI: 1.40–6.41), and for herds having more than 50 animals (OR of 11.0; 95% CI: 3.75–32.46). *Brucella abortus* biovar 3 was isolated from the hygromas.

**Conclusion:**

brucellosis in Niger is a serious problem among cattle especially in the rural areas around Niamey and among sheep in the urban areas of Niamey. The seroprevalence varies across strata and animal species with important risk factors including herd size, abortion and transhumance at herd level and age at animal population level. For effective control of brucellosis, an integrated approach seems appropriate involving all stakeholders working in public and animal health.

## Introduction

Worldwide, brucellosis remains an important disease in humans, domestic and wild animals [1]. It is an infectious disease caused by bacteria of the genus *Brucella* which comprises eight species ranked according to their pathogenicity and host preferences. Six of the eight species can be isolated from terrestrial mammals: *B. abortus, B. melitensis, B. suis, B. canis, B. ovis et B. neotomae* [2]. The disease is endemic in sub-Saharan Africa (SSA), with significant effects on economic and social conditions of people in this region [3]. Indeed, brucellosis has an important impact on the health and productivity of livestock greatly reducing their economic value [4]. The epidemiology of brucellosis in SSA is complex and the prevalence varies across geographic regions and livestock systems [5]. The disease incidence is influenced by management factors, herd size, population density, type of animal breed and biological features such as herd immunity [6], [7], [8], [9], [10], [11]. In West Africa, the rates of infection vary greatly from one country to another, within a country and production systems [12], [13], [14], [15], [16]. It is generally accepted that the prevalence of brucellosis is much higher in the pastoral grazing systems than the urban and periurban systems where herd sizes are smaller [5], [10], [17], [18], [19].

In Niger, brucellosis was first reported in 1953 in humans [20], but it was not until 1970 that the first preliminary serological studies were conducted to assess the prevalence of the disease in animals [21]. There are few data on human brucellosis in West Africa, particularly in Niger [5], [6], [7], [24]. Gidel et al. [21] showed seroprevalence rates ranging from 1% to 17% in humans in pastoral areas of Côte d'Ivoire, Niger and Burkina Faso. According to the same authors, the prevalence of the disease in 1974 was 0.5% in the city of Niamey [21]. Since then, very little research has been conducted in order to assess the magnitude of, and risk factors for the disease transmission within different production systems. Later, investigations in pastoral livestock systems of the country in 1986 by Akakpo et al. [12], Akakpo and Bornarel [22], and in 1991 by Bloch and Diallo [13] have confirmed the presence of brucellosis in cattle with apparent prevalence rates ranging between 1.4% and 30.9%.

The increased demand for animal products following the growth of the urban population and the depletion of food resources in pastoral areas due to climate change is forcing livestock keepers and their animals to move to the peripheral cities [23]. This has led to the development of a dynamic and complex livestock production system in the urban and suburban regions of Niamey city [24]. Breeders are in most cases installed on unhealthy and unmanaged land without adequate infrastructure to conduct their activities [25]. Dietary habits of Niger population such as consumption of unpasteurized dairy products, close contact with infected herds and with contaminated environmental sources could be major risk factors for the spread of *Brucella* infections among humans [24], [25], [39], [43]. The contribution of these and other factors to the epidemiology of brucellosis in livestock production systems in Niger is not yet known.

The aim of this study was to determine the prevalence of *Brucella* infection, using indirect Enzyme-linked Immunosorbent assay (iELISA) in cattle, goats and sheep in the urban, periurban and surrounding rural areas of Niger and to identify risk factors for infection both in human and livestock populations. In addition, we used some hygroma fluid to identify a field circulating strain of *Brucella*.

## Materials and Methods

### 2.1. Ethics statement

This study involves a questionnaire based survey of farmers as well as blood sampling from their animals. The study protocol was assessed and approved by the Niger National Advisory Committee on Ethics with reference number 010/2009/CCNE and by the Ministry of Agriculture and Livestock of the Republic of Niger with reference number 00109 on 28 January 2010. Participants provided their verbal informed consent for animal blood sampling as well for the related survey questions, according to the Niger procedures at the time of the study. Collection of blood samples was carried out by professional veterinarians adhering to the regulations and guidelines on animal husbandry. In each village, a meeting was held with the community members to explain the purpose of the study. Farmers were not forced to participate in the survey and animal blood sampling. Name, region and village of the farmers were registered. Paper questionnaires were encoded and recorded in Excel and names were replaced by their coded versions for analysis. Paper questionnaires were stored in Niger.

### 2.2. The study area

**Figure 1:**
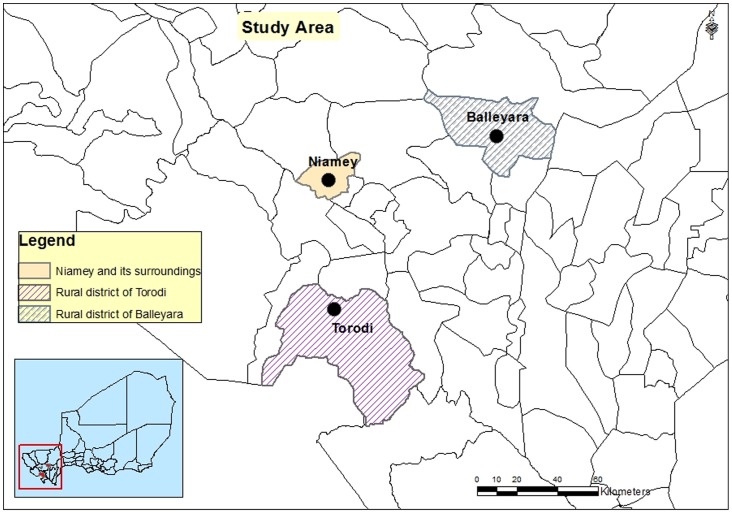
Location of the study areas in Niger.

The study zone was composed of three strata in accordance with the classification established by Boukary et al. [24]: the urban (Ur), the periurban (Pu) of Niamey and the rural areas (Ru) (**Fig. 1**).

The urban area was formed by the Urban Community of Niamey (UCN) located along the Niger River in the western part of the country, between 2° 10′ and 2° 14′ longitude E and 13° 33′ and 13° 36′ latitude N and covered an area of about 12,500 ha with nearly one million inhabitants.

The periurban area covers a ray ranging from 5 to 25 km around the capital. It is populated by the long-established resident population and a population of immigrants composed mainly of Fulani herders. The installation of the latter was promoted by the development of the dairy industry and the increase in demand for milk in the capital [24]. They occupy makeshift homes generally subjected to inadequate measures of sanitation and hygiene. Their animal breeding strategy consists in keeping only lactating females and genitor males in the sites. The renewal of the animals is done from the main transhumant herd located mostly in rural areas of Balleyara and Torodi [24].

For the rural area, the community of Balleyara located about 110 km northeast of the capital and the community of Torodi located 80 km southwest of Niamey at the border with Burkina Faso and Benin were considered as are the main rural poles which supply the city of Niamey with cattle, small ruminants and animal products.

Studying the interactions between rural, urban and surrounding rural areas through various exchange relationships between people and their herds seem very interesting in understanding the mechanisms of transmission of zoonotic diseases such as brucellosis and justifies the inclusion of this rural strata in the present study.

### 2.3. Study design and data collection

The study took place between December 2007 and October 2008 within the three strata previously defined and was conducted in two phases. First, a cross-sectional household survey was carried out and secondly, blood sampling and hygroma fluid collection were performed on animals belonging to herds led by the households surveyed. These samples were used for laboratory analysis.

#### 2.3.1 The cross-sectional household survey

Since the study area was divided into three strata; urban, peri-urban and surrounding rural areas of Niamey, the first step was to identify the number of sampling sites. A total of 45 sampling sites were randomly selected from a roster of 375 sites identified within the three strata. In each study stratum, the approximate number of herds (which belonged to different sites) was listed with the assistance of local veterinary officers and farmers' leaders. The total number of herds to be included in this study was calculated using an expected herd level seroprevalence “*p*” of 14.2% [22], a confidence level of 95%, desired absolute precision (*d*) of 0.05 and using the following formula 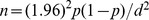 [26]. This yielded a total of 187 herds to be sampled from each strata. However, since herds turn to be similar within sites, a correction factor of magnitude 2 [27] was applied to account for the clustering of herds within sites. In addition, contingencies were adjusted for by adding another 25% of herds leading to a total of 234 herds to be sampled from each strata. The sampling of the herds within sites was based on a proportionate sampling scheme since the total number of herds within each site was available. All animals present at the time the herd was visited were sampled.

In this study, herd means all animals reared within the household surveyed (i.e., ecosystem) and it was regarded as the primary sampling unit according to the study area. So, there were as many herds as households surveyed.

The questionnaire used in the face-to-face interview with the head of the selected households included questions related to risk factors for transmission of brucellosis both in animals and humans. At the animal level, information was collected on species (goats, sheep, cattle), age (in years), and gender (male or female). At the herd level, the factors included: herd size (number of animals in the household), occurrence of abortion (yes/no), whereas relative to the household, the factors were: practices related to livestock (acquisition modes of the animals by the household, method of rearing animals, handling of newly arrival animals, fate of dead animals or aborted foetuses), and the social status of the household (native of the locality or migrant). The full questionnaire in French is available as supporting information document (see questionnaire S1).

#### 2.3.2. Blood sample collection and testing

Five thousand one hundred and ninety-two (5,192) serum samples and sixteen (16) hygroma fluid samples were collected from animals (Table 1). The blood sample collection was made during the face-to-face interviews with the head of the household.

**Table 1:** Total number of herds surveyed and animals tested in the urban (Ur), peri-urban (Pu) and rural areas (Ru) of Niger.

Variable	Ur	Pu	Ru	Total
***Data on herds surveyed***				
* - Number of sites indentified*	19	131	225	375
* - Number of sites selected*	9	13	23	45
* - Number of herds (households interviewed)*	239	215	227	681
***Data on animals tested***				
* - Cattle*	973	1,473	724	3,170
* - Sheep*	216	320	650	1,186
* - Goats*	106	150	583	839
Total number of animals tested	1,295	1,943	1957	5,195

The collected samples were stored in a deep freezer (−20°C) at the National Reference Laboratory for AIDS and Tuberculosis (NRL-HIV/TB) of Niamey (Niger), until they could be analysed at the National Reference Centre for Brucellosis, Veterinary and Agrochemical Research Centre (CODA-CERVA) in Belgium. All assays except MLVA performed at CODA-CERVA are accredited (ISO 17025).

#### 2.3.3. Serological testing

For procedural reasons, our samples were sent to Belgium 2 years after collection. Serological tests were conducted between September 2009 and February 2010. An indirect ELISA described previously by Limet et al. [28] was used. The antigenic use in this test is a purification of the lipopolyssacharide of *Brucella abortus* W99. Briefly, 50 µl of serum dilutions (1∶50 in buffer consisting of 0.1 M glycine, 0.17 M sodium chloride, 50 mM EDTA, 0.1% (volume) Tween 80, and distilled water, pH 9.2) were added to the wells in duplicate. The plates were incubated for 1 h at room temperature. Binding of antibodies was detected using a protein-G peroxydase conjugate (Biorad, Belgium). The conjugate was incubated for 1 h at room temperature. Citrate–phosphate buffer containing 0.4% o-phenylenediamine and 2 mM H2O2 was used to visualize the peroxydase activity. The difference in optical densities (OD) at A 490 and 630 nm was read on a Bio Kinetics Reader EL-340 (Biotek Instruments, Vermont, USA). Negative control serum and dilution buffer was added in duplicates on each plate as controls. This ELISA fulfils the requirement laid down in the OIE Manual of Standards for Diagnostic Tests and Vaccines [1].

#### 2.3.4. Bacteriological testing

Directly after 15 minutes centrifugation at 3000 rpm, isolation of *Brucella*sp. from Hygroma was performed according to the technique described by Alton et al [29] and Bankole [30]. Isolate of *Brucella* were typed by classical method and molecular method (MLVA). A 15 locus VNTR typing was carried out according to Le Flèche et al. [31]. The 15 loci have been classified into two panels, panel 1 (eight minisatellite loci) and panel 2 (seven microsatellite loci) (Table 2). The profile obtained from the MVLA was compared to other strain profiles using MVLA Public Databases (MLVAbank 2012).

**Table 2:** Loci of the Variable Number Tandem Repeats analysis (VNTR) used in the study (according to [31]).

Panel	Reference VNTR a	Name of marker b
1	BRU1322_134bp_408bp_3u	Bruce06
	BRU1134_18bp_348bp_4u	Bruce08
	BRU211_63bp_257bp_3u	Bruce11
	BRU73_15bp_392bp_13u	Bruce12
	BRU424_125bp_539bp_4u	Bruce42
	BRU379_12bp_182bp_2u	Bruce43
	BRU233_18bp_151bp_3u	Bruce45
	BRU2066_40bp_273bp_3u	Bruce55
2	BRU1543_8bp_152bp_2u	Bruce04
	BRU1250_8bp_158bp_5u	Bruce07
	BRU588_8bp_156bp_7u	Bruce09
	BRU548_8bp_152bp_3u	Bruce16
	BRU339_8bp_146bp_5u	Bruce18
	BRU329_8bp_148bp_6u	Bruce21
	BRU1505_8bp_151bp_6u	Bruce30

Legend;

a reference VNTR: naming nomenclature includes repeat unit size, PCR product size in strain 16 M, corresponding repeat copy number,

b common name of the marker.

### 2.4. Statistical analysis

#### 2.4.1. Determination of the true prevalence of brucellosis

The estimation of the true prevalence (TP) of brucellosis at the animal population level was done using the formula proposed by Rogan and Gladen [32]: 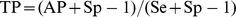
where AP is the apparent prevalence; Se is the sensitivity and Sp is the specificity.

Because no prior data were available for Niger, the specificity (Sp) and sensitivity (Se) of the iELISA were the values of the study carried out on traditional livestock farming systems in Ivory Coast by Thys et al. [14]. The values of Se and Sp for the iELISA and their 95% confidence intervals based on this study were as follows: 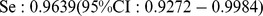


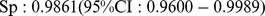



A herd was considered positive if at least one animal tested positive for *Brucella* antibodies by the iELISA test within the herd. The animal population and herd-level AP were estimated using an intercept-only random effects logistic regression model with site and herd as random effects to account for the survey design characteristics of the study. Treating herd and site for the animal population level analysis and site as a random effect in the herd-level analysis as random effects accounted for the clustering of animals within herds and the clustering of herds within sites respectively. In addition, it accounted for differences in number of animals within herds and number of herds within sites. For the different sub-populations such as male or female cattle, sheep and goat within each stratum, the AP and TP were computed using random effects logistic regression models. Normal 95% confidence intervals were computed for both the AP and the TP.

#### 2.4.2. Risk factor analysis

The risk factor analysis was performed at the animal and herd/household levels. It should be noted that the data on the herd are combined and processed together with data on the household. In what follows, the ‘herd level’ denotes the analysis of factors collected at the household and herd-level. In addition, the animal population level analysis was done separately for cattle and small ruminants. Initially, a univariate analysis was performed using a univariate random effects logistic regression model at the animal population level as well as at the herd level. The animal population level model used as response, the brucellosis status of the animals and each animal level risk factor or indicator variable in turn as explanatory variables whereas the herd level model used as response, the herd level brucellosis status and corresponding herd level risk/indicator factors as covariates.

For the animal population level analysis, herd and site location were used as random effects to account for potential clustering of animals within herds (dependence of results from the same herd) and clustering of herds within sites whereas site location was used as a random effect for the herd level analysis to account for the effects of clustering of herds within sampling sites. At the animal population level and at the herd level, the variable representing the three strata was forced into the model to account for variations in prevalence across strata.

Variables with a p-value<0.10 in the univariate analysis were further evaluated in a multivariable random effects logistic regression analysis. A manual forward stepwise selection approach was applied to choose the final model. In the first step of this approach, univariate models were built for each covariate. The best univariate model was selected based on the AIC values (the smaller the better). The remaining variables were then added each in turn to the best univariate model to form two-variable models. The best two-variable model was selected as that with the smallest AIC among the two-variable models. This procedure was repeated until the addition of one more variable failed to improve the model fit; in other words once the AIC started to increase or remained constant. The model with the smallest AIC was considered to be the most appropriate model for the data.

The effects of confounding were investigated by observing the change in the estimated odds ratios of the variables that remain in the model once a non-significant variable is removed. When the removal of a non-significant variable led to a change of more than 25% of any parameter estimate, that variable was considered a confounder and was not removed from the model.

Multicollinearity was assessed among the independent variables using the Cramer's phi prime statistic which expressed the strength of the association between two categorical covariates. Values >0.7 were indicative of co-linearity and in this case only the variable most significantly associated with the response was kept in the model [35].

All two-way interaction terms of the variables remaining in the final model were assessed for significance based on the likelihood ratio test comparing the model with the desired interaction term and the corresponding model with no interaction terms.

The intra-class correlation coefficient (ICC), which is a measure of the degree of clustering of animals belonging to the same herd or of herds belonging to the same site, was computed. In random effects logistic regression models, the individual level variance 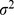 on the logit scale is usually assumed to be fixed to 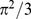 [34]. The variability attributed to differences between herds was given by: 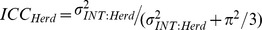
whereas that between sites was computed as: 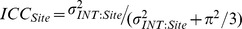



If the ICC is low or zero in either case, it implies that the animals within herds or the herds within sites are independent (there is no clustering) of each other and therefore random effects should not be included in the analysis. On the contrary an ICC close to 1 implies that there is high between herds or between site heterogeneity implying the clustering of individual animals within herds or clustering of herds within sites respectively [35].

The models were built using the xtmelogit function in STATA, version 12.1, software (SataCorp LP, College station, Texas). Model selection was done using Laplacian approximation whereas parameter estimates from the final model were obtained using Adaptive Gaussian Quadrature [36]. The robustness of the final model was assessed by increasing the number of Quadrature (integration) points and monitoring changes in parameter estimates [37].

## Results

### 3.1. Herd structure

A total of 5,195 animals composed of 3,170 cattle, 1,186 sheep and 839 goats were sampled. These animals belonged to 681 herds which in turn were nested within the 45 sites (9 in the urban region, 13 in the peri-urban region and 23 in the rural area). The number of herds reduced from 702 to 681 because of incomplete information for 21 of the sampled herds. Regardless of region of origin it was found that the herds were mixed and included the three species: cattle, sheep and goats. However, in the urban and peri-urban areas, cattle were the most numerous. They were respectively 72% and 78% of the herds which showed that these farms were mainly oriented to dairy production. In rural pastoral areas, herds were more balanced with 38% of cattle, 33% sheep and 29% goats (**Table 1**). The different cattle breeds included: Azawak, Mbororo, Djelli, Goudali and crossbred and were found to be widely distributed across strata. For small ruminants, the common breeds for sheep were Oudah (Bali Bali) and Ara Ara whereas for goats the common breed was Sahel. All were also found to vary widely across strata.

### 3.2. *Brucella* seroprevalence and potential risk factors

#### 3.2.1. Brucella seroprevalence results

Of the 5,195 sera examined, 2.6% tested positive for iELISA (137/5195, 95% CI: 2.2–3.1%). The estimated overall animal population-level true prevalence in the study population was 1.3% (95% CI: 1.1–3.4) (**Table 3**). The prevalence of brucellosis was highly variable among the animal species considered.

**Table 3:** Apparent prevalence (AP) and estimated true prevalence (TP) of brucellosis at the individual animal and herd levels.

		Urban						Periurban						Rural					
Species	Gender	N	Positive	Apparent Prevalence (%)		True estimated Prevalence* (%)		N	Positives	Apparent Prevalence (%)		True estimated Prevalence* (%)		N	Positive	Apparent Prevalence (%)		True estimated Prevalence* (%)	
				AP	95% CI	TP	95% CI			AP	95% CI	TP	95% CI			AP	95% CI	TP	95% CI
**Cattle**	Female	908	26	2.0	1.1–2.9	0.6	0.1–3.4	1337	32	1.8	1.1–2.6	0.5	0.1–2.9	649	36	4.4	2.8–5.9	3.1	1.8–7.3
	Male	65	1	1.0	0.0–3.3	0	-	136	3	1.5	0–3.6	0.1	0–4.1	75	5	5.3	0.2–10.4	4.1	0.0–14.9
	Total	973	27	2.0	1.1–2.9	0.6	0.1–3.4	1473	35	1.8	1.2–2.5	0.5	0.1–2.9	724	41	4.6	3.1–6.2	3.4	2.1–7.5
**Sheep**	Female	206	10	4.1	1.4–6.8	2.8	0.6–9.0	274	3	0.8	0.0–1.9	-	-	575	17	2.5	1.2–3.7	1.1	0.3–4.6
	Male	10	0	-	-	-	-	46	0	-	-			75	0	-	-	-	-
	Total	216	10	3.6	1.1–6.1	2.3	0.3–8.1	320	3	0.6	0.0–1.5	-	-	650	17	2.1	1.0–3.2	0.8	0.1–3.9
**Goats**	Female	95	1	0.8	0.0–2.7	-	-	133	0	-	-	-	-	535	3	0.4	0.0–1.7	-	-
	Male	11	0	-	-	-	-	17	0	-	-	-	-	48	0	-	-	-	-
	Total	106	1	0.7	0.0–2.2	-	-	150	0	-	-	-	-	583	3	0.4	0.0–0.9	-	-
**Herd**	Total	227	42	13.5	9.1–18.0	12.8	8.4–22.3	215	27	12.0	7.7–16.4	11.2	7.0–20.6	239	33	17.8	12.9–22.6	17.2	12.5–27.4

Legend: * According to the formula proposed by Rogan and Gladen [30].

Brucellosis prevalence varied according to strata. In cattle, it was significantly higher in rural areas with a true prevalence (TP) of 4.6% (95% CI: 3.1–6.2) against 2.0% and 1.8% in urban and peri-urban areas respectively. For small ruminants, the prevalence of brucellosis also varied across strata even though differences were not statistically significant i.e. the 95% confidence intervals overlapped. In sheep, the overall true prevalence of brucellosis was 3.6% (95% CI: 1.1–6.1) in urban areas where it is higher than in periurban and rural areas (**Table 3**).

At the herd level, the estimation of the true prevalence of brucellosis across the three strata indicated that 91 out of 681 herds investigated (13.7%) were found to be maintaining infected animals (Table 3). The true herd-level prevalence (THP) of brucellosis ranged between 11.2% and 17.2% according to the area in consideration.

#### 3.2.2. Potential risk/indicator factors associated with sero-prevalence of brucellosis based on univariate random effects logistic regression analysis

The results of the univariate analysis which was based on random effects models correcting for animal level clustering indicated that at the animal population level, age was significantly associated with brucellosis seropositivity for cattle (P<0.05) (**Table 4**). In general, it was observed that the prevalence of brucellosis was significantly higher in older animals compared to young animals since their confidence intervals do not overlap. Animals aged between 1 and 4 years appeared more at risk than young animals and animals older than 4 years.

**Table 4:** Potential risk/indicator factors associated with individual animal-level brucellosis seropositivity among 5195 animals nested within 681 herds.

Variable	Number tested (Positive)	% Positive (95% CI)	Odds ratio (95% CI)	P-value
***Cattle***				
**Strata**				**0.003**
*Periurban*	1473 (35)	2.4(1.7–3.3)	1 (Ref.)	
*Urban*	973 (27)	2.8(1.8–4.0)	1.3 (0.54–2.67)	
*Rural*	724 (41)	5.7(4.1–7.6)	2.8 (1.37–5.60)	
**Age (years)**				**<0.001**
≤*1*	912 (16)	1.8 (1.0–2.8)	1 (Ref.)	
*>1 and <4*	1307 (61)	4.7(3.6–6.0)	3.7 (1.87–7.17)	
*≥4*	951 (26)	2.7(1.8–4.0)	1.7 (0.83–3.68)	
**Gender**				0.944
Bull	276 (9)	3.3 (1.5–6.1)	1 (Ref.)	
Cow	2894 (94)	3.2 (2.6,4.0)	1.1 (1.53–2.36)	
***Small ruminants (sheep and goats)***				
**Strata**				**0.018**
*Peri-urban*	470 (3)	0.6 (0.1–1.9)	1 (Ref.)	
*Urban*	322 (11)	3.4 (1.7–6.0)	5.4 (1.41–20.88)	
*Rural*	1233 (20)	1.6 (1.0–2.5)	2.4 (0.68–8.56)	
**Age (years)**				**0.161**
≤*1*	318 (4)	1.3 (0.3–3.2)	1 (Ref.)	
*>1 and <4*	723 (12)	1.7 (0.9–2.9)	1.3 (0.55–3.14)	
*≥4*	984 (18)	1.8 (1.1–2.9)	2.1 (0.79–5.69)	
**Gender**				**0.026**
Male	207 (0)	0.0 (0–1.8)	1 (Ref.)	
Female	1818 (34)	1.9 (1.3–2.6)	8.0 (0.94–131.35)^exact^	

Exact: estimates based on Firth's logistic regression model; Ref: reference group.

Among small ruminants, the effects of gender could not be evaluated using the random effect logistic regression model, because there were no positive cases among males. However, a univariate analysis was performed using Firth's logistic regression analysis. Firth's logistic regression analysis was used in place of the traditional exact logistic regression analysis to overcome the computational limitations and convergence issues caused by the sparseness (separation) of the data. The method uses penalized maximum likelihood (PML), which is carried out iteratively until model convergence to estimate the associated odds ratios, standard errors, and 95% confidence intervals [38]. The results indicated that gender was not significantly associated with brucellosis seropositivity among small ruminants but since the p-value was <0.10 it was considered as a potential risk factor to be included in the multivariable analysis (**Table 4**).

The univariate random effects logistic regression analysis with a random effect for site and a fixed effect for strata, revealed that the herd level risk factors: herd composition, transhumance, abortion in the herd, acquisition of animals, handling of newly arrived animals, herd size and origin of herds, all appeared to be highly significantly associated with the herd level brucellosis sero-positivity (P<0.05) (**Table 5**).

**Table 5:** Potential risk factors associated with herd level seroprevalence of brucellosis based on a univariate random effects model with strata forced in as a fixed effect and site as a random effect.

Variable code	Level	Odds ratio (95% C.I)	P-value
Herd Composition	Animal species that occur within the herd belonging to the herd surveyed		<0.001
	*1: Cattle*	1(Ref.)	
	*2: Cattle + Sheep or Goat*	4.8(1.20–19.46)	
	*3: Sheep or Goat*	3.3(0.92–12.00)	
	*4: Cattle + Sheep + Goat*	8.9(2.58–30.90)	
			
Herd size	Number of animals owned by the herd		<0.001
	1:< = 10	1(Ref.)	
	2:>10 and < = 50	3.3(1.27–8.40)	
	3:>50	27.9(9.9–78.7)	
Abortion	Presence of females who aborted among the animals belonging to the herd surveyed		<0.001
	*1: No*	1 (Ref.)	
	*2: Yes*	4.5(2.23–8.95)	
Acquiring animals	Acquisition modes of the animals by the herd		0.025
	*1: Heritage*	1 (Ref.)	
	*2: Fostering*	1.2(0.34–4.69)	
	*3: Purchase*	1.7(0.80–3.72)	
	*4: Mix*	2.7(1.32–5.65)	
Transhumance	Method of rearing animals of sedentary type *(not migratory : No)* or nomadic *(transhumant : Yes)*		<0.001
	*1: No*	1(Ref.)	
	*2: Yes*	9.1(5.06–16.30)	
Handling	Handling of newly arrival animals *(mixed with other animals or quarantined)*		0.022
	*1: Quarantined*	1 (Ref.)	
	*2: Mixed*	1.8(1.08–2.85)	
Native	Origin of the herd surveyed : native of the locality *(Yes)* or migrant *(No)*		<0.001
	*1: Yes*	1(Ref.)	
	*2: No*	4.3(2.15–8.64)	

Sero-prevalence: Having or not at least one animal testing positive by Elisa-test within the herd (1 or 0). Strata: Stratum in which the investigations took place *(Urban, Periurban, Rural). Site:* Means the village, hamlet or the district selected for the study within the different strata. Herd: Herd surveyed within the different sites. Ref.: reference group.

### 3.3. Multiple random effects logistic regression model

The results of the multivariable random effects logistic regression analysis at the animal-population level indicated that for cattle, the variables representing strata and age were important risk factors whereas for small ruminants, only the variable representing strata was found to be important (**Table 6**). On the other hand, out of the 8 potential risk factors initially considered in the multiple random effects logistic regression model only transhumance, abortion in the herd and herd size) were included in the final herd level model (**Table 7**). None of the two-way interaction terms were statistically significant (p>0.05). No evidence of confounding was present and the estimated Cramer's phi prime statistic values were all less than 0.7 indicating no important correlations between the independent variables. Increasing the number of quadrature points had no influence on the estimated fixed effects and the variance component parameters indicating that the models were robust.

**Table 6:** Final model of animal population level risk factors associated with brucellosis seropositivity among cattle and small ruminants.

Variable code	Level	Odds ratio (95% C.I)	P-value
	**Cattle**		
Strata	*Periurban*	1(Ref.)	
	*Urban*	1.4(0.73–2.62)	0.323
	*Rural*	2.8(1.48–5.17)	0.003
			
Age (years)			
	≤*1*	1(Ref.)	
	*>1 and <4*	2.7(1.43–5.28)	0.002
	*≥4*	1.2(0.59–2.60)	0.527
***Random effects***		***SE(95% CI)***	
Herd level variance	1.20	0.45(0.57–2.50)	
	**Small ruminants**		
Strata	*Periurban*	1 (Ref.)	
	*Urban*	5.5(1.48–20.38)	0.011
	*Rural*	(0.70–8.50)	0.161
***Random effects***		***SE(95% CI)***	
Herd level variance	0.26	0.42(0.01–6.13)	

Legend: Ref.: reference group.

**Table 7:** Final model of herd-level risk factors associated with brucellosis sero-positivity among 681 herds which nested within 45 sites.

Variable code	Level	Odds ratio (95% C.I)	P-value
Strata	Stratum in which the investigations took place		
	*Periurban*	1(Ref.)	
	*Urban*	1.5(0.37–6.25)	0.334
	*Rural*	1.8(0.55–5.70)	0.557
Herd size	Number of animals in the herd		
	< = 10	1 (Ref.)	
	>10 and < = 50	1.9(0.71,5.15)	0.199
	>50	11.0(3.75,32.46)	<0.001
Abortion	Presence of females who aborted among the animals belonging to the herd surveyed		
	*No*	1(Ref.)	
	*Yes*	3.0(1.40–6.41)	0.005
Transhumance	Method of rearing animals of sedentary type *(not migratory : No)* or nomadic *(transhumant : Yes)*		
	*No*	1(Ref.)	
	*Yes*	5.4(2.84–10.41)	<0.001
***Random effects***		***SE***	***95% CI***
Site level variance	1.69	0.68	(0.77–3.72)

Legend: Ref.: reference group.

The variance components of the final model for cattle indicated that the ICC for herd was 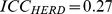 and for small ruminants 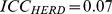. The substantial ICC for cattle implies that there is considerable between-herd heterogeneity and thus clustering of animals within herd whereas for small ruminants the low ICC implies that the animals within herd are independent (there is no clustering). The ICC for the herd-level data was 0.34 suggesting that there is considerable clustering of herds within sites. The considerable cattle-level clustering and herd-level clustering demonstrates the potential for herd-level and site-level interventions to influence brucellosis seropositivity.

From the final model for cattle (**Table 6**), it can be seen that the odds of brucellosis sero-positivity were significantly higher in rural areas as compared to periurban areas with an OR of 2.8. In addition, for cattle between 1 and 4 years old the odds of brucellosis seropositivity were 2.7 times higher compared to those that are 1 year old. For small ruminants, the odds of brucellosis seropositivity were significantly higher in urban areas as compared to periurban areas with an OR of 5.5.

At herd level, the final multivariable model (**Table 7**) yielded that for households that reported the presence of abortions in the herd, the odds of seropositivity were 3 times higher as compared to households which did not report the occurrence of abortions. Also for herds that reported the practice of transhumance, the odds of sero-positivity were 5.4 times higher compared to those that did not practice transhumance. Finally, for herds with more than 50 animals, the odds of brucellosis seropositivity were 11 times higher compared to herds with less than 10 animals.

### 3.4. Strain typing and identification

**Figure 2:**
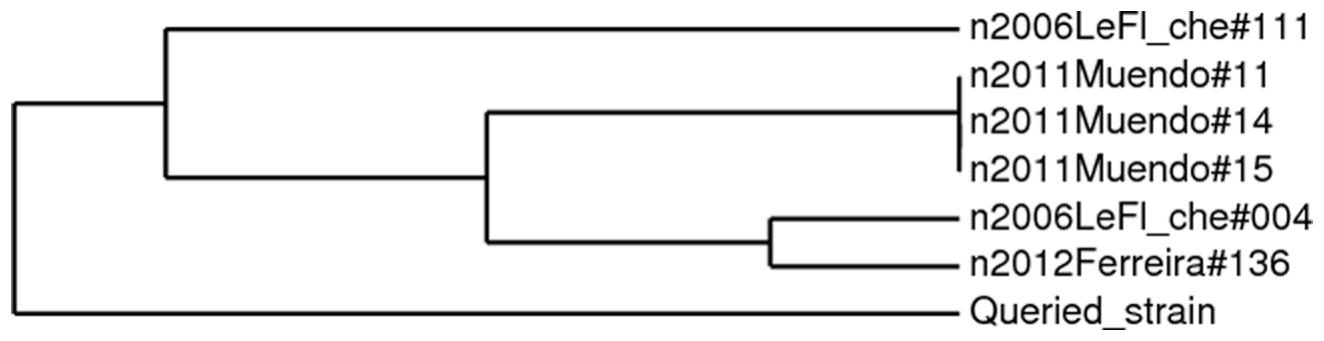
Clustering analysis of a field strain of *Brucella abortus* 3 from Niger (Queried_Strain) with field and reference strains in the *Brucella* multiple loci variable number tandem repeats analysis (MLVA) database (MVLABANK, 2012) using panels 1 and 2. The data are given in columns from left to right: year of isolation and ‘alias’.

Of the 16 hygroma samples collected and cultured, only one was positive after 3 days of incubation and showed round (1–2 mm diameter), convex colonies with entire edges and smooth shiny surfaces. Colonies required CO2 for growth, produced H_2_S and grew in the presence of basic fuchsin, thionin and safranin. The determination of biotype was based on the results of four tests: hydrogen sulphide production, agglutination by monospecific anti-A and anti-M sera, growth in the presence of dyes, and carbon dioxide requirement. The profile of this isolate was classified as *B. abortus* biovar 3, according to the Corbel and Brinley-Morgan [50] classification. The number of tandem repeats for each locus is shown in **Table 8**. Considering only the first panel, this profile appeared to be related to *B. abortus* biovar 3 reference strain Tulya and dromedary strain BCCN 93_26 from Uganda (Le Flèche_2006). This type is also close to *B. abortus* biovar 3 strain BCCN 93_26 from Sudan (Le Flèche_2006), *B. abortus* biovar 3 strain 11-KEBa2, 14-KEBa2 and 15-KEBa2 from Kenya (Muendo_2011) and *B. abortus* biovar 3 reference strain Tulya (Ferreira_2012). The relationship between these strains and our isolate is shown in **Fig. 2**.

**Table 8:** The Multiple Loci Variable Number Tandem Repeats analysis (MLVA) profiles showing number of variable tandem repeats (VTR) for *a B. abortus* biovar 3 isolate from Niger (Queried Strain) and its closest MLVA neighbour profile.

	Strain	REF Tulya	BCCN 93–26	11-KEBa2	14-KEBa2	15-KEBa2	REF Tulya	Queried Strain
	Host	human	dromedary	cattle	cattle	cattle	cattle	cattle
	Publication	Le Flèche 2006	Le Flèche 2006	Muendo 2011	Muendo 2011	Muendo 2011	Ferreira 2012	This study
	Country	Uganda	Sudan	Kenya	Kenya	Kenya	-	Niger
**VTR**	bruce06	3	3	3	3	3	3	3
	bruce08	5	5	5	5	5	5	5
**Panel 1**	bruce11	4	4	4	4	4	4	3
	bruce12	11	11	11	11	11	11	11
	bruce42	2	2	2	2	2	2	2
	bruce43	2	2	2	2	2	2	2
	bruce45	3	3	3	3	3	3	3
	bruce55	3	3	3	3	3	3	3
**VTR**	bruce18	8	6	7	7	7	8	8
	bruce19	40	40	40	40	40	42	21
**Panel 2**	bruce21	8	8	8	8	8	8	8
	bruce04	6	6	6	6	6	6	6
	bruce07	5	8	5	5	5	5	2
	bruce09	3	3	3	3	3	3	3
	bruce16	11	7	12	12	12	11	12
	bruce30	5	7	5	5	5	5	7

## Discussion

The study confirms that brucellosis is present in Niger and that herd level seroprevalence varied by abortion status of the herd, herd size and method of rearing animals.

Due to lack of unbiased data and standardized method to estimate the seroprevalence in Niger and across the West African sub-region, comparing our findings with those from other studies should be made with caution. The apparent prevalence in our study was low compared with that obtained in other studies conducted in Niger. Indeed, using the Rose Bengal Test (RBT), Akakpo et al. [12] found an AP rate of brucellosis of 27.7% in the Kirkissoye ranch not far from Niamey, while Bloch and Diallo [13] reported an AP rate ranging from 3.7% to 9.5%. Using RBT, Boukary et al. [39] reported an AP rate of brucellosis comprised between 2.4 and 5% in smallholder dairy cattle herds in the urban and periurban areas of Niamey. The difference in prevalence between our study and the previous ones may be partly explained by the methodology used in the study protocol. In fact, in some studies, the lack of sampling frames or their imperfection does not allow to achieve representative sampling [40]. Another important issue is the difference in sensitivity and specificity of serological tests used for screening. This factor contributes to the variability of results among researchers [5], [6], [41], [42]. The reported high prevalence in the other studies might be due to false-positive serum reactions [10]. The RBT used for screening individual animals at national-local-level is cheap, rapid and highly sensitive [1]. However, its specificity is low because the smooth lipopolysaccharides of the *Brucella* antigen can cross-react with antibodies from closely related Gram-negative bacteria such as *Yersinia enterocolitica* O∶9, *Escherichia coli* O∶157, *Salmonella spp*., and *Sternotrophomonas maltophilia* as well as antibodies produced by *B. abortus* S19 vaccine [41], [42].

The fact that the risk of transmission of brucellosis in animals at the population and herd level varied significantly depending on the strata is in agreement with the findings of several authors who demonstrated variations in the prevalence of brucellosis related to the production systems [5], [7], [15], [16], [18]. In cattle, we found that that the risk of brucellosis seropositivity was higher in rural areas compared to periurban and urban areas. The reason for the higher prevalence in the rural areas was probably due to the fact that in this area, free animal movement is common [24], [39]. It is now well documented that the dynamics and frequent migration of pastoral herds might increase the chance of coming into contact with other potentially infected herds and exposure to geographically limited or seasonally abundant diseases [7], [11], [12], [16], [43]. Considering the contagious nature of *Brucella* species, sharing grazing land and drinking water facilitate transmission of the disease [8], [9], [10]. Another factor that may explain the high sensitivity of cattle to *Brucella* spp. in rural areas is linked to the herd composition. We also observed in that area that the herds are equally mixed, while in the urban and periurban areas cattle are more abundant than sheep and goats. Although the factor “herd composition” was not retained in our final model, our results based on a univariate random effects model showed that the risk of contamination increases sharply in mixed herds where the odds of brucellosis seropositivity was 8.9 times higher compared to pure cattle herds. This is in accordance with Holt et al. [33] and Megersa et al. [11].

Considering the strata, the odds of testing positive to brucellosis in cattle were significantly higher in the rural areas than in urban ones. This can be explained by the difference in management. The Fulani of the periurban area of Niamey developed a new strategy for dairy production which involves keeping only animals in production (dairy cows in early stage of gestation or in lactation) the rest of the herd being kept in rural areas [24]. The low prevalence of brucellosis in cattle in periurban results from this strategy as only apparently healthy animals are selected for milk production [43]. This is in agreement with our observation that the seroprevalence of brucellosis increased with the incidence of abortions. Indeed, the odds of seropositivity were 3.0 times higher in the herds where the presence of abortions was reported as compared to those which did not report the occurrence of abortions. This is in accordance with several authors who found that the prevalence of brucellosis within herds is positively correlated with the incidence of abortions in females [6], [44], [45].

Contrary to what we observed in cattle, the risk of infection with brucellosis in small ruminants was much higher in urban compared to rural and periurban areas. Indeed, the odds of brucellosis seropositivity were 5.4 times higher in urban compared to periurban areas for small ruminants. Difference in management can also explain this, as small ruminants play a very important economic role in urban areas. For many households, keeping sheep and goats is a way of saving money [25]. Males are kept separately where they are fed with forage complemented and with kitchen waste. Their market value is much higher than that of females and they are usually sold when there is a need for cash or are slaughtered during religious ceremonies [23]. This explains the low number of males in the samples used in our study and also their low susceptibility to brucellosis infection. Unlike rural areas where herds are usually mixed, urban flocks are in most cases separated from cattle. Ewes and she-goats of the different flocks are typically collected by a shepherd who brings them to the pasture [24], [25]. These specific conditions of raising small ruminants in urban areas promote aggregation of animals within neighborhoods, pastures and water points, favouring the transmission of the disease [11], [43], [45].

Transhumance in Niger is much more pronounced in pastoral areas where large herds have to run long distances searching for pasture and water points [12], [21], [24], [25]. We observed that the risk of contracting the disease increases significantly in herds with high mobility. The odds of seropositivity were 9.1 times higher in these herds compared to those that did not practice transhumance. Similarly, our results showed that herd size was linked to *Brucella* seropositivity (P<0.001) and that the risk of contamination was much higher in larger herds compared to those with a limited number of animals. These results corroborated those of several other authors [6], [10], [40], [46]. In Niger, the history of migration is closely linked to that of transhumance. Under pressure from repeated drought and deterioration of their livelihoods, pastoralists tend increasingly to become sedentary [25]. These people usually are installed on marginal lands where sanitation and hygiene infrastructures are generally lacking [24]. The absence of veterinary services brings these migrants to assist themselves pregnant or aborted females [24]. This will expose them to a higher risk of dissemination and transmission of the disease.

In our study, the prevalence of brucellosis in cattle was highly correlated with the age of the animals. Indeed, for cattle between 1 and 4 years old, odds of brucellosis seropositivity were 3.7 times higher compared to those that are 1 year old or younger. That higher seropositivity of animals between 1 and 4 years old could be explained due to the increase in exposure [12], [18], [47], [48], [49]. Indeed these animals are more mobile and therefore more exposed to infection by *Brucella* within the transhumant herds. Animals less than one year old are generally kept in the household together with lactating females.

At the household level, our results showed that mixing of newly arrived animals into the herd is highly correlated with brucellosis seropositivity. Females infected with *Brucella spp*. excrete high concentrations of the organism in their milk, placental membranes and aborted foetuses [51]. Therefore, there is a high risk of transmission of the pathogen between animals and from animals to humans through direct contact with contaminated material such as foetal membranes, aborted foetuses and other animal products. According to an investigation conducted in the periurban and rural areas of Niger by Boukary et al. [43], it seems that due to the lack of veterinary services, farmers assist in delivering cows without gloves or masks, which puts them at high risk of infection with *Brucella*.

Our study aimed also to identify strains of *Brucella spp* circulating in Niger. Out of the 16 hygroma samples, one sample was found positive after culture and *Brucella abortus* biovar 3 was isolated. We were not able to isolate *Brucella* from the remaining 15 samples analyzed, although they were collected from animals tested positive for iELISA. This can be explained by the fact these samples would probably not contain enough germs to allow their isolation. Another reason is that the shelf life and transport conditions of samples may have a negative effect on the survival of *Brucella*. Indeed our hygroma samples were collected between December 2007 and October 2008. They were kept during 2 years at −20° C prior to shipment to Belgium, where they were analyzed. Possible electric power outages during storage, thawing of samples during transport and handling may have affected the quality of the hygroma liquid. The difficulty of isolating *Brucella* from hygroma fluids under similar conditions to ours has already been mentioned by Bankole et al. [30].

Our *Brucella* isolate shows the same characteristics as those already isolated in Niger by Akakpo et al. [12]. In fact, strains of *Brucella abortus* isolated in Africa are known to grow slowly, to be sometimes negative on the oxidase test and to have a specific oxidative pattern [53]. This finding is similar to the results obtained by several authors in West and Central Africa who reported the presence of *B. abortus* biovar 3 or intermediate 3/6 [12], [22], [30], [40], [46]. So, *Brucella abortus* biovar 3 is very common in Africa. Considering the panels of MLVA profile, our isolate profile appeared to be related to *B. abortus* biovar 3 strains isolated in Uganda and Sudan [31] and those isolated in Kenya [54].The profile also appeared to share some similarities with *B. abortus* biovar 3 reference strain Tulya isolated by Ferreira et al. [55].

In conclusion, the present study confirms the existence of *Brucella* in cattle, sheep and goats from the three studied strata. It highlights the presence of *Brucella abortus* biovar 3 and stresses that age, practice of transhumance, herd size and occurrence of abortions are risk factors for the spread of the disease within animals. These risk factors are related to the complexity of interactions that exist within and between the different production systems and the different practices observed in urban, periurban and rural areas.

At present, there is no officially coordinate program control of brucellosis in Niger. The role played by the disease in limiting livestock production and its economic impact on the livestock industry in Niger has not yet been evaluated. Attitudes of communities have to be defined regarding the brucellosis, the feasibility and the acceptability of potential measures. Measures including selective vaccination programme in herds with high prevalence combined with the slaughtering of known infected animals *(test and slaughter)* in herds with low infection rates as well as testing animals newly introduced into the herd can be considered [52], [56]. For effective control of this disease in the context of sub-Saharan Africa, an integrated approach should be promoted that takes into account the relationship between humans, animals and environment. A multisectorial framework involving physicians, veterinarians, and all the stakeholders working in public and animal health in the context of a “One Health” approach is recommended.

## Supporting Information

Questionnaire S1 **Questionnaire for the cross-sectional household survey on animal husbandry practices and eating habits among rural livestock keepers.**
(DOC)
