# Supplementary material for: Seroprevalence and Potential Risk Factors for Brucella Spp. Infection in Traditional Cattle, Sheep and Goats Reared in Urban, Periurban and Rural Areas of Niger
Source: PLoS One. 2013 Dec 16;8(12):e83175. doi: 10.1371/journal.pone.0083175 (PMC3865157; doi:10.1371/journal.pone.0083175)
Supplement: Questionnaire S1 — Questionnaire for the cross-sectional household survey on animal husbandry practices and eating habits among rural livestock keepers. (DOC) [file pone.0083175.s001.doc]

#

#### **Nom de l’enquêteur ____________________________________________________________**

#### **Nom de la personne interrogée __________________________________________________________________**

#### **Date d’entretien ______________________ heure début ________________ heure fin ____________________**

####

#### **Nom du site __________________________** Code localité _________________________

**Distance par rapport à Niamey_________________ Coordonnées géographiques**

**S1 questionnaire for the cross-sectional household survey on animal husbandry practices and eating habits among rural livestock keepers (French)**

**I- INFORMATIONS SUR LE MENAGE**

###### A/ Identification

1. **Nom du chef de ménage** **:** _______________________________________________
2. **Ethnie :** 1. Peul 2. Zarma 3. Haoussa 4.  Arabe 5. Touareg 6. Autres _________
3. **Sexe :** 1.  Masculin 2.  Féminin
4. **Age : ________________**
5. **Niveau d’instruction :** 1. Aucun 2. Alphabétisé 3. Primaire 4. Instruit (niveau BEPC) 5. Coranique
6. **Situation matrimoniale :** 1.  Marié (nbre de femmes ____) 2.  Veuf/ve

3.  Divorcé 4.  Célibataire 5.  Autre ________________

1. **Quelles sont par ordre d'importance les activités du ménage qui occupent le plus du temps à la famille?** *(numéroter les réponses par ordre d’importance par a, b, c, d...*

1.  Elevage ____ 2.  Agriculture ____ 3.  Artisanat _____

4.  Commerce ____ 5.  Exode ____ 6.  Autres (à préciser) : ___________________

1. **Composition du ménage à charge selon les classes d'âges**

Mettre dans chaque case correspondante l’âge de l’individu concerné

|  | **Hommes** | **Femmes** | **Filles** | **Garçons** |
| --- | --- | --- | --- | --- |
| 1 |  |  |  |  |
| 2 |  |  |  |  |
| 3 |  |  |  |  |
| 4 |  |  |  |  |
| 5 |  |  |  |  |
| 6 |  |  |  |  |
| 7 |  |  |  |  |
| 8 |  |  |  |  |
| 9 |  |  |  |  |
| 10 |  |  |  |  |

1. **Comment sont réparties les différentes activités liées à l’élevage au sein du ménage?**

**Faire une croix dans la case correspondante et noter le temps (en heures) que prend chaque activité**

| **Activités**  **Catégories** | **Conduite au pâturage** | **Abreuvement** | **Traite** | **Transformation du lait**  **(caillage, …)** | **Vente du lait** | **Autres**  **(préciser)** |
| --- | --- | --- | --- | --- | --- | --- |
| Filles |  |  |  |  |  |  |
| Garçons |  |  |  |  |  |  |
| Femmes |  |  |  |  |  |  |
| Hommes |  |  |  |  |  |  |
| Autres |  |  |  |  |  |  |

###### B/Hygiène de l’habitat *(A noter par l’enquêteur après observations)*

1. **Etat de salubrité du site**

1.  Bon *(pas d’égout=effluents, pas d’eau stagnante, bien balayé)* 2.  Moyen

3.  Mauvais 4.  Très mauvais *(présence d’égouts, de saletés avec difficultés de se déplacer)*

1. **Etat de salubrité du ménage**

1.  Bon *(cour bien balayée, différents matériels de ménage ou de travail (matériel de traite, vente du lait, … ) lavés et bien rangés à l’abris de poussière)*

2.  Moyen

1.  Mauvais

4.  Très mauvais *(présence d’égouts, de déjections humaines ou animale, de saletés avec difficultés de se déplacer)*

1. **Où sont parqués les animaux ?**

1.  Dans la concession 2.  Dans la rue

3.  En dehors du site 4.  Autre :_____________________________________

1. **L’enclos des animaux est –il clôturé ?**

 Oui  Non

Si Oui : noter le type clôture :______________

Noter son état actuel :  Bon *(les animaux ne peuvent pas sortir)*  Mauvais *(autrement)*

1. **Etat de salubrité de l’enclos des animaux**

1.  Bon *(Balayé, paillé, pas de liquides et déjections en dehors de l’enclos)*

2.  Moyen

 Mauvais (les animaux sont sales et baignent dans les déjections et saletés avec difficultés de se déplacer)

###### C/Les relations entre le milieu rural et urbain

1. **Avez vous un ou plusieurs membres de votre famille (ou vous-même) qui séjourne de manière régulière ou temporaire à Niamey ou dans les environs?**  Oui  Non

*(Si non passer directement à la question 24 au II. Informations sur le troupeau ….)*

1. **Si oui :**
   - 1. quel est le but de ce déplacement ?

1.  Exode 2.  Vente d’animaux sur pied

3.  Vente du lait 4.  Visite _____4.  Autre________________________________

- - 1. Nom du site ou quartier d’accueil :___________________________________________
    2. Nom et adresse (N° téléphone, boîte postale, …) du responsable de votre famille (personne ressource) :________________________________

1. **Comment s’effectue le déplacement des personnes entre votre village d’origine et votre site ou votre quartier d’accueil à Niamey ?**

Remplir le tableau suivant en tenant compte des spécifications suivantes :

- Pour la Période, utiliser les abréviations suivantes : **SSC =** Saison Sèche Chaude ; **SSF**= Saison Sèche Froide ; **SP**: Saison des Pluies

| **Catégorie** | **Nombre** | **Période** | **Durée du séjour (en mois)** | **Motif du déplacement** |
| --- | --- | --- | --- | --- |
| Vieux (non actif) |  |  |  |  |
| Hommes |  |  |  |  |
| Femmes |  |  |  |  |
| Garçons |  |  |  |  |
| Filles |  |  |  |  |

1. **Par quels moyens de déplacement de ces personnes vont-elles ?**

1.  A pied 2.  En voiture 3.  Autre :__________________________________

1. **Comment s’effectue le déplacement des personnes entre votre site ou quartier d’accueil à Niamey  et votre village?**

Remplir le tableau suivant en tenant compte des mêmes spécifications que dans la question précédente

| **Catégorie** | **Nombre** | **Période** | **Durée du séjour (en mois)** | **Motif du déplacement** |
| --- | --- | --- | --- | --- |
| Vieux |  |  |  |  |
| Hommes |  |  |  |  |
| Femmes |  |  |  |  |
| Garçons |  |  |  |  |
| Filles |  |  |  |  |

1. **Par quels moyens de déplacement de ces personnes viennent-elles ?**

1.  A pied 2.  En voiture 3.  Autre :__________________________________

**II- INFORMATIONS SUR LE TROUPEAU**

1. **Quand et à quelle fréquence s’effectue le déplacement des animaux entre votre village et votre site ou quartier d’accueil à Niamey ?**

Remplir le tableau suivant en tenant compte des spécifications suivantes :

- Pour la Période, utiliser les abréviations suivantes : **SSC =** Saison Sèche Chaude ; **SSF**= Saison Sèche Froide ; **SP**: Saison des Pluies

|  | **Catégorie** | **Nombre** | **Période** | **Durée du séjour au niveau du site** | **Motif du transfert**  (vente, production laitière, embouche, don, …) | **Mode de transport utilisé**  (voiture, à pied, …) |
| --- | --- | --- | --- | --- | --- | --- |
| B  O  V  I  N  S | Femelles L. |  |  |  |  |  |
|  | Jeunes |  |  |  |  |  |
|  | Mâles adultes |  |  |  |  |  |
| O  V  I  N  S | Femelles L. |  |  |  |  |  |
|  | Jeunes |  |  |  |  |  |
|  | Mâles adultes |  |  |  |  |  |
| C  A  P  R  I  NS | Femelles L. |  |  |  |  |  |
|  | Jeunes |  |  |  |  |  |
|  | Mâles adultes |  |  |  |  |  |

1. **Comment s’effectue le déplacement des animaux entre votre site ou quartier d’accueil à Niamey et votre village?**

Remplir le tableau suivant en tenant compte des mêmes spécifications que dans la question précédente

|  | **Catégorie** | **Nombre** | **Période** | **Durée du séjour au niveau du site** | **Motif du transfert**  (vente, production laitière, embouche, don, …) |
| --- | --- | --- | --- | --- | --- |
| B  O  V  I  N  S | Femelles L. |  |  |  |  |
|  | Jeunes |  |  |  |  |
|  | Mâles adultes |  |  |  |  |
| O  V  I  N  S | Femelles L. |  |  |  |  |
|  | Jeunes |  |  |  |  |
|  | Mâles adultes |  |  |  |  |
| C  A  P  R  I  NS | Femelles L. |  |  |  |  |
|  | Jeunes |  |  |  |  |
|  | Mâles adultes |  |  |  |  |

1. **Par quels moyens de déplacement les animaux sont transportés ?**

1.  A pied 2.  En voiture 3.  Autre :__________________________________

1. **Quelles sont les modalités d’acquisition du troupeau par le ménage ?** (*Préciser par ordre d'importance en utilisant les lettres a, b, c…)*

1.  Achat ____ 2.  Confiage ____ 3.  Héritage ____ 4.  Don ____

5. Habbanayé ____ 6.  Autres (à préciser) _____________________________

1. **Etes-vous seul propriétaire de votre bétail ?** . 1 Oui 2.  Non

1. **Si non avec qui êtes-vous associés** :

1.  Membre de la famille 2.  Même tribu 3.  Même région 4.  Autre région

5.  Autres (à préciser) _____________________________

1. **Quelle est la composition actuelle de votre troupeau ?**

| **Catégorie** | **Bovins** | **Ovins** | **Caprins** |
| --- | --- | --- | --- |
| Nombre Femelles adultes en production |  |  |  |
| Nombre Jeunes femelles |  |  |  |
| Nombre Jeunes mâles |  |  |  |
| Nombre Mâles adultes |  |  |  |
| Autres |  |  |  |

1. **Quelles sont les principales races que vous élevez par espèce ?**

| **Espèces** | **Races** | **Raisons** |
| --- | --- | --- |
| **Bovines** | 1. **_____________________** 2. **_____________________** 3. **_____________________** 4. **_____________________** | 1. **_____________________** 2. **_____________________** 3. **_____________________** 4. **_____________________** |
| **Ovines** | 1. **_____________________** 2. **_____________________** 3. **_____________________** | 1. **_____________________** 2. **_____________________** 3. **_____________________** |
| **Caprines** | 1. **_____________________** 2. **_____________________** 3. **_________________** | 1. **_____________________** 2. **_____________________** 3. **_________________** |

**III- INFORMATIONS SUR LES PRATIQUES D’ELEVAGE**

1. **Comment est conduite la reproduction des animaux ?**
2.  Accouplement libre (non contrôlé)
3.  Accouplement des femelles reproductrices avec un mâle sélectionné
4.  Autre :__________________________________________________
5. **Comment sont choisissez-vous les animaux que vous élevez ?**

1.  Pour leur production laitière 2.  Résistance aux maladies 3.  Résistance aux conditions difficiles (alimentation) 4.  Bonne production de viande (aptitude à l’embouche) 5.  Autres (à préciser) _____________________________

1. **Quelles sont les mesures prises lors de l’introduction d’un nouvel animal**

1.  L’animal est d’abord tenu à l’écart pour observation. Pendant quelle durée :______________ 2.  L’animal est directement mélangé avec les autres animaux du troupeau

3.  Autre :__________________________________

1. **Qui fait la traite ?_________________________________________________**
2. **Combien de temps dure la traite (minutes)__________________________________**
3. **Avant la traite, est-ce que le matériel est nettoyé ?** 1 Oui 2.  Non

Si oui avec quoi ?

1.  de l’eau + eau de javel 2.  de l’eau simple 3.  de l’eau + détergent (OMO) 4.  de l’eau bouillie 5.  Autre :__________________________________________________

1. **Après la traite, est-ce que le matériel est nettoyé ?** 1 Oui 2.  Non

Si oui avec quoi ?

1.  de l’eau + eau de javel 2.  de l’eau simple 3.  de l’eau + détergent (OMO) 4.  de l’eau bouillie 5.  Autre :__________________________________________________

1. **Quelles sont les maladies apparues dans votre troupeau ces dernières années (types de maladie, les mesures préventives et curatives)**

| **Type de maladie** *(nom vernaculaire ou symptômes)* | **Mesures préventives prises  par l'éleveur** | **Mesures curatives prises  par l'éleveur** |
| --- | --- | --- |
|  |  |  |
|  |  |  |
|  |  |  |
|  |  |  |
|  |  |  |
|  |  |  |
|  |  |  |

1. **Quelles sont les précautions prises pour éviter la maladie ?**

1.  vaccination 2.  aucune mesure 3.  Autre :________________________________

1. **Que faites-vous avec le cadavre des animaux qui meurent ?**

1.  Enterrer 2.  Brûler 3.  Jeter dans une poubelle

4.  Autre :__________________________________________________

1. **Que faites-vous des avortons en cas d’avortement ?**

1.  Enterrer 2.  Brûler 3.  Jeter dans une poubelle

1.  Autre :__________________________________________________
2. **Que faîtes-vous du placenta en cas d’avortement ?**

1.  Enterrer 2.  Brûler 3.  Jeter dans une poubelle

 Autre :__________________________________________________

**IV. HABITUDES ALIMENTAIRES**

1. **Vous arrive-t-il d’abattre des animaux vous même ? 1 Oui 2.  Non**

Si oui :

a) où se fait l’abattage :

1.  dans la cour de la maison 2.  devant la porte de la maison 3.  autre :____________________________

b) Quels types d’animaux sont abattus:__________________________________________________

c) Pour quelle raison?

1.  Consommation familiale 2.  Vente 3.  Don

4.  Autre :__________________________________________________

1. **Qui fait la dépouille en cas d’abattage ?__________________________________**
2. **Qui fait la découpe ?__________________________________________________**
3. **Vous arrive-t il de manger ou de servir de la nourriture pendant vous manipulez de la viande sans vous laver les mains ?**

1 Oui 2.  Non

1. **Sous quelle forme consommez vous la viande ?**

1.  Grillée 2.  Bouillie 3.  Séchée

4.  Autre :__________________________________________________

1. **Consommez vous du lait ? 1 Oui 2.  Non**
2. **De quelle espèce animale ?**

1.  Vache 2.  chèvre 3.  Brebis

4.  Chamelle 5.  Autre :__________________________________________________

1. **sous quel type le lait est consommé ?**

1.  Lait frais 2.  Lait frais Caillé 3.  Lait bouilli

4.  Lait en poudre 5.  Autre :__________________________________________________

1. **Consommez-vous d’autres produits laitiers à base du lait cru ?**

1.  Doonou 2.  Tchoukou 3.  Autre :________________________________

1. **Vous arrive-t il de manger ou de servir de la nourriture pendant vous manipulez du lait cru ou caillé sans vous laver les mains ?**

1 Oui 2.  Non

**V. INCIDENCE DE LA TB ET DE LA BRUCELLOSE**

1. **Avez-vous dans votre troupeau des animaux qui toussent actuellement ?**

1 Oui 2.  Non

Si oui préciser :

- L’espèce animale : 1.  Bovins 2.  Ovins 3.  Caprins
- Le sexe : 1 Mâle 2.  Femelle
- La catégorie : 1 jeune 2.  adulte

1. **Depuis quand (noter le moment) :** ____________________________________________

1. **Quels traitements avez-vous fait :**_____________________________________________

**_____________________________________________________________________________**

1. **Avez des animaux qui étaient morts de toux persistante ?** 1 Oui 2.  Non
2. **Aviez vous traitez ces animaux ?** 1 Oui 2.  Non
3. **Si oui, avec quoi ?**_________________________________________________________
4. **Avez des animaux qui maigrissent malgré un déparasitage et une alimentation conséquente (qui gardent leur appétit) :** 1 Oui 2.  Non
5. **Y a-t-il dans votre entourage des gens (une ou plusieurs personne) souffrant de toux persistante avec amaigrissement ?** 1 Oui 2.  Non
6. **Avez-vous souffert ou souffrez vous actuellement de toux persistante ?**

1 Oui 2.  Non

1. **Si oui, aviez-vous été dans les centres de soins de santé ou à l’hôpital.**

1 Oui 2.  Non

-Si non, pourquoi ?____________________________________________________________

-Si oui, quels sont les soins qu’on vous a donnez et examens que vous aviez faits :__________

____________________________________________________________________________

1. **Y a-t-il dans votre troupeau des animaux qui poussent des cris répétitifs sans raison apparente ?** 1 Oui 2.  Non

Si oui, depuis combien de temps : __________________________________________________

1. **Avez-vous observé des cas d’avortement chez les femelles de vos animaux d’élevage ?**

1 Oui 2.  Non

Si oui préciser:

| **Espèce** | **Durée de la gestation au moment de l'avortement (mois)** | **Période de l'année  (SP, SSC, SF)** |
| --- | --- | --- |
| Vache |  |  |
| Chèvre |  |  |
| Brebis |  |  |

1. **Dans votre troupeau, avez-vous observé des mâles qui ont des problèmes aux testicules ?**

1 Oui 2.  Non

Si oui :

| **Espèce** | **Nombre** | **Période de l'année  (SP, SSC, SF)** |
| --- | --- | --- |
| Taureau |  |  |
| Bouc |  |  |
| Bélier |  |  |

1. **Avez-vous observé des avortements chez les femmes dans votre entourage ?**

1 Oui 2.  Non

Si oui :

- Pendant quelle période ? _________________________________________________
- A quelle fréquence ?_____________________________________________________

1. **Souffrez des fièvres chroniques (prolongés ou ondulantes) et rebelles aux traitements ?** 1 Oui 2.  Non
2. **Vous arrive t-il de suer abondamment la nuit ?**

1 Oui 2.  Non

1. **Connaissez-vous quelqu’un dans votre entourage ou dans votre famille qui présente ces symptômes**

Si oui : depuis quand ?_______________________________________________
